# Enhancing Data-Driven Decision-Making in HIV Care With Viral Load and Early Infant Diagnosis Data Dashboards in Côte d’Ivoire: Qualitative Study

**DOI:** 10.2196/76550

**Published:** 2026-03-31

**Authors:** Yao He, Yves-Rolland Kouabenan, Paul Henri Assoa, Nancy Puttkammer, Stephen Gloyd, Noah Hoffman, Bradley H Wagenaar, Casey Iiams-Hauser, Pascal Komena, N’zi Pierre Fourier Kamelan, Adama Sanogo Pongathie, Jan Flowers, Nadine Abiola, Natacha Kohemun, Jean-Bernard Koffi Amani, Christiane Adje-Toure, Lucy A Perrone

**Affiliations:** 1 Digital Initiatives Group, International Training and Education Center for Health, Department of Global Health, Schools of Public Health and Medicine, University of Washington, Seattle, WA, United States; 2 International Training and Education Center for Health- Côte d'Ivoire (I-TECH CIV), Abidjan, Côte d’Ivoire; 3 Department of Global Health, University of Washington, Seattle, WA, United States; 4 Department of Laboratory Medicine and Pathology, University of Washington, Seattle, WA, United States; 5 Department of Epidemiology, Schools of Public Health and Medicine, University of Washington, Seattle, WA, United States; 6 Direction de l'Informatique et de la Santé Digitale Sanitaire, Ministère de la Santé, de l’Hygiène Publique et de la Couverture Maladie Universelle, Abidjan, Côte d’Ivoire; 7 Division of Global HIV and TB, United States Centers for Disease Control and Prevention, Abidjan, Côte d’Ivoire; 8 Department of Pathology and Laboratory Medicine, University of British Columbia, 2211 Wesbrook Mall, Vancouver, BC, V6T 1Z7, Canada, 1 604 827 2927

**Keywords:** health data dashboards, data-driven decision making, data dashboard, data visualization, user experience, HIV/AIDS

## Abstract

**Background:**

Data dashboards are popular tools for supporting routine monitoring and decision-making in public health. Two dashboards were developed in Côte d’Ivoire to visualize laboratory data on HIV viral load (VL) and early infant diagnosis (EID) testing.

**Objective:**

This study assessed the attitudes and experiences regarding data-driven decision-making and the VL and EID dashboards among existing and potential dashboard users in Côte d’Ivoire.

**Methods:**

We conducted a qualitative study including 2 focus group discussions (FGDs) and 12 in-depth interviews (IDIs). The conceptual framework for the use of health data in decision-making guided the FGDs, and the Consolidated Framework for Implementation Research informed the IDIs. We used deductive and inductive approaches to analyze the interview data.

**Results:**

The 26 participants were from 17 organizations; 11 (42.3%) were female. The participants reported a supportive data culture that valued data-driven decision-making and external pressure from the United States President’s Emergency Plan for AIDS Relief (PEPFAR) that motivated data use. The dashboards were considered useful for monitoring performances and making decisions for service delivery and laboratory operations. Existing users used the dashboards regularly. Potential users expressed interest in the speed and ability to track progress. The participants considered the dashboards simple and straightforward compared to other analytical tools but suggested updating the dashboards more frequently and visualizing more data.

**Conclusion:**

The study highlighted the importance of supportive data culture and the potential of dashboards to promote data use. However, challenges such as limited access to the internet and equipment for potential users need to be addressed.

## Introduction

Progress around HIV/AIDS prevention and treatment has stagnated around the world [1], and the HIV response needs to be reinvigorated to achieve the 95-95-95 goals set by the Joint United Nations Programme on HIV/AIDS (UNAIDS) [2]. HIV viral load (VL) suppression and early infant diagnosis (EID) are important health and service delivery outcomes for monitoring timely and effective treatment, preventing transmissions, and assessing prevention of mother-to-child transmission. Using data and evidence strengthens the decision-making by country leadership and contributes to successful HIV responses [3]. To help achieve the targets for VL suppression and EID in Côte d’Ivoire based on high-quality data, the national VL and EID data dashboards provide information that supports the monitoring, evaluation, and decision-making in HIV-related services and programs.

Understanding the context, culture, and behavior around data demand and use is important to ensure the effectiveness of the tools that aim to support data-driven decision-making, especially in low- and middle-income countries (LMICs). According to a 2023 literature review, the main barriers to data-driven decision-making in LMICs include insufficient data use competencies, low data quality, inadequate data availability, lack of systems design and user-centered design, disconnect between data producers and users, unsupportive leaders, absence of organizational supports, misalignment in data needs between donors and local stakeholders, weak data use culture, and low individual motivation [4]. Evidence is limited to whether and how these barriers influence data-driven decision-making in HIV responses in Côte d’Ivoire, and such evidence will provide useful context for understanding the successes and issues with the VL and EID dashboards.

Dashboards are tools designed to support data-driven decision-making by visualizing quantitative data and supplying audit and feedback to decision makers and service providers [5,6]. Applications of dashboards are ubiquitous in public health, with purposes ranging from disease surveillance [7-9] and monitoring of quality and efficiency [10-14] to clinical decision support [15-18] and stock management, among others [19,20].

Despite the proliferation of dashboards in public health, studies that assess data needs and user experience of dashboard users are limited. After reviewing 1191 papers, the authors of a systematic review on public health dashboards identified only 18 user studies, while most studies describe only the design and technical development process of a specific dashboard [21]. Only a few of the 18 user studies considered data demands and self-efficacy of dashboard users [21], highlighting a notable gap in research that other reviews already identified previously [22,23].

This study aimed to fill the research gaps described above by answering the following questions: What were the attitudes and experiences of existing and potential dashboard users on data-driven decision-making in their organizations? What were the participants’ impressions of the VL and EID dashboards?

## Methods

### Intervention Description

To respond to the need for a tool that consolidates and presents data in an accessible format, the publicly accessible, online dashboard for VL was developed in 2017 through the collaboration among the International Training and Education Center for Health (I-TECH), the Côte d’Ivoire Ministry of Health (MOH), the United States President’s Emergency Plan for AIDS Relief (PEPFAR), the United States Centers for Disease Control and Prevention (CDC), The United States Agency for International Development, the Clinton Health Access Initiative, and the Kenyan MOH [24,25]. I-TECH obtained the software source code in the HTML for the Kenyan VL dashboard and adapted it for the Ivorian context to visualize monthly aggregate VL data from the open-source enterprise-level laboratory information system (OpenELIS; Digital Initiatives Group at the University of Washington [26]), the electronic laboratory information system in Côte d’Ivoire [27,28]. The visualization tool was Highcharts, and the data sources were monthly OpenELIS datasets at the laboratory level. The use case was to provide analyzed, critical information for monitoring program performance and progress toward achieving the goals for viral load testing and suppression and EID. The target users were the CDC and the PEPFAR implementing partners, policy makers at different levels of the MOH in Côte d’Ivoire, and clinical and laboratory service providers.

Data visualizations produced by the VL dashboard included bar charts and pie charts for the number of tests, turnaround time, and viral suppression with disaggregation by gender, age group, and reasons for seeking care. All charts except for the national average turnaround time could be disaggregated by month, region, district, clinical laboratory, or PEPFAR implementing partners. Users could access the disaggregated visualizations through the navigation tabs at the top of the dashboard webpage. A dedicated tab showed charts about viral suppression by gender, age, reason for the test request, and antiretroviral regimen. All data visualizations, source datasets, and a dashboard user guide were globally accessible and available for download.

In 2020 the EID dashboard was created based on the VL dashboard to display monthly aggregate EID data from OpenELIS [29]. The types of visualizations and functions are similar to those described above for the VL dashboard. More details about the development process, information visualized, and types of visualizations of the VL and EID dashboards were described elsewhere [24].

### Study Design and Data Collection

We conducted a qualitative study including 2 focus group discussions (FGDs) and 12 semistructured in-depth interviews (IDIs). Specifically, the FGDs were intended to gather general perspectives that the participants converged or diverged on about data-driven decision-making and the VL and EID dashboards. The IDIs were intended to probe for greater depth of response with themes that arose in the FGDs. Data collection took place from September 2021 to April 2022 through in-person or online meetings.

The study population consisted of existing and potential users of the VL and EID dashboards. Existing users were staff members of national and regional clinical laboratories, the CDC office in Côte d’Ivoire, and PEPFAR implementing partners. Potential users were from MOH divisions and clinical laboratories that reportedly had not used the dashboards, but the I-TECH project team and existing users thought these entities would find the dashboards useful.

To enroll existing users, we purposefully sampled individuals who were already using the dashboards based on previous meetings to participate in the first FGD and subsequent IDIs. We then used snowball sampling to ask the FGD participants to introduce us to other existing users from additional organizations to participate in the IDIs.

To enroll potential users, we first purposefully sampled a staff member of the Programme National de Lutte contre le Sida (PNLS; National AIDS Control Program) who had shared with the study team that they had not used the dashboards. The study team also identified individuals who had not used the dashboards and were heads or deputy heads of the regional clinical laboratories in Côte d’Ivoire. We purposefully chose PNLS because, as the national technical group created by the MOH to guide the national HIV/AIDS response, PNLS knew about the dashboards but reported that they did not routinely use them. Regional laboratories might also be interested in the dashboards because they were referral sites that processed all VL and EID samples referred from other laboratories in the region. We invited identified potential users to participate in the second FGD and subsequent IDIs. Recruitment stopped once we confirmed that all targeted types of organizations were represented and that thematic saturation had been reached.

The conceptual framework for the use of health data in decision-making (referred to as the “data use framework” hereafter) informed the interview guides for the 2 FGDs [4,30]. The data use framework builds on the Performance of Routine Information System Management (PRISM) framework created by the MEASURE Evaluation group [4,30,31]. The framework highlights key activities that most directly influence data demand and use and subsequently the building blocks in the health system and ultimately health outcomes. We chose to focus on the activities that would influence data demand and use to see if and how they were reflected in the participants’ responses.

The relevant constructs of the Consolidated Framework for Implementation Research (CFIR) informed the interview guides for the IDIs (Table 1) [32,33]. The CFIR is a meta-theoretical framework designed to explain the factors that affect translation of interventions into practice and their sustainment in practice across different contexts [32,33].

The interview guides were initially developed in English and later translated into French. The FGDs and IDIs were recorded, and comprehensive notes were taken. On average, the FGDs lasted 59 minutes, and the IDIs lasted 32 minutes. The recordings were transcribed verbatim in French and translated into English for analysis.

A combination of deductive and inductive approaches was used to analyze the data. Codes were created using the activities that influence data demand and use from the data use framework, the CFIR constructs, as well as those derived from the data. All transcripts were coded by one primary coder (YH). YH first chose one FGD transcript and one IDI transcript to code with the initial codebook that consisted of the deductive codes. After adding inductive codes to the codebook, YH revised the coding in the first two transcripts and continued coding the rest of the transcripts. Any subsequent changes in the codebook or coding approach were applied to transcripts that had been coded previously. We summarized the coded information into themes that corresponded to the research questions. The two frameworks served as the basis for the themes, with additional themes derived from the data that did not fit the constructs of the two frameworks. Two additional team members (YRK and PHA) reviewed the coded data and thematic summaries to validate the analysis and ensure consistency in interpretation. The coding and thematic analysis were conducted in ATLAS.ti (8 Windows; ATLAS.ti Scientific Software Development GmbH).

**Table 1.** The *a priori* components of interest from the conceptual framework for the use of health data in decision-making and the Consolidated Framework for Implementation Research (CFIR).

Data collection method	Conceptual framework for the use of health data in decision-making^a^	CFIR constructs^b^
FGD1^c^: existing dashboard users FGD2: potential dashboard users	Data use context Data demand and use infrastructure	None.
		
IDIs^d^ with individual existing or potential dashboard users	Data use context Data demand and use infrastructure Data users and data producers Data quality	Innovation Relative advantage Adaptability Design Inner setting Access to knowledge and information Outer setting External pressure – market pressure Policies and laws Financing Individuals Capability

a Also referred to as the data use framework.

b Both the 2019 and 2022 versions of the CFIR were referenced.

c FGD: focus group discussion.

d IDI: in-depth interview.

### Ethical Considerations

This study was determined to be nonhuman participants research by the University of Washington Institutional Review Board and approved by Côte d’Ivoire Comité National d’Ethique des Sciences de la Vie et de la Santé (CNESVS, Ivorian Institutional Review Board; reference number 006‐21/MSHP/CNESVS-km). All participants were unpaid volunteers and provided written informed consent. To ensure data confidentiality, the data collected were stored in a project-specific, encrypted instance of Google Drive managed by the secure server environment of the University of Washington that is compliant with the US Health Insurance Portability and Accountability Act (HIPAA) guidelines.

## Results

### Overview

We conducted one FGD and 7 IDIs with existing users and another FGD and 5 IDIs with potential users. There were 26 participants across the FGDs and IDIs; 11 (42.3%) were female; 17 organizations were represented (Table 2). The participants from the CDC and PNLS were program officers; the participants from the PEPFAR implementing partners were clinical program leads or strategic information team members; and the participants from national or regional referral laboratories were heads or deputy heads of the laboratories.

**Table 2.** Characteristics of the study participants who were existing or potential users of the HIV viral load and early infant diagnosis data dashboards in Côte d’Ivoire in 2021‐2022.

Characteristics	Overall	FGD1^a^ with existing users	FGD2 with potential users	IDIs^b^ with existing users	IDIs with potential users
Participants from each type of organization, n (%)	26 (100)	9 (34.6)	5 (19.2)	7 (26.9)	5 (19.2)
US CDC^c^	3 (11.5)	2 (22.2)	1 (20.0)	0 (0.0)	0 (0.0)
PEPFAR^d^ IP^e^	9 (34.6)	7 (77.8)	0 (0.0)	2 (28.6)	0 (0.0)
PNLS^f^	1 (3.8)	0 (0.0)	0 (0.0)	0 (0.0)	1 (20.0)
National referral laboratory	6 (23.1)	0 (0.0)	1 (20.0)	4 (57.1)	1 (20.0)
Regional referral laboratory	7 (26.9)	0 (0)	3 (40.0)	1 (14.3)	3 (40.0)
Organizations represented (mins)	17	6	5	6	5
Participants by sex, n (%)					
Female	11 (42.3)	3 (33.3)	2 (40.0)	2 (28.6)	4 (80.0)
Male	15 (57.7)	6 (66.7)	3 (60.0)	5 (71.4)	1 (20.0)
Mean recording length in minutes	36	70	48	36	26

a FGD: focus group discussion.

b IDI: in-depth interview.

c US CDC: United States Centers for Disease Control and Prevention.

d PEPFAR: United States President’s Emergency Plan for AIDS Relief.

e IP: implementing partner.

f PNLS: Programme National de Lutte contre le Sida (National AIDS Control Program).

### Data-Driven Decision-Making

#### Data Use Context

According to all the participants in the FGDs and IDIs, their organizations used data in decision-making, and there was no difference between the organizations of the existing users and those of the potential users. Not only would the leaders use data in decision-making, but different units and levels within each organization also used data in their work, even if some units or levels were not seen as decision makers. Data were used routinely to monitor technical and operational performance as well as ad hoc under special circumstances to assess the situation, identify problems or needs, and make decisions to solve problems or make improvements. The most common areas where the participants used data to monitor were laboratory operational processes (eg, nonconformity of samples, sample flows, and turnaround time) and lost-to-follow-up clients for VL testing.

#### Data Demand, Data Use Infrastructure, Data Users, and Data Producers

The FGD with existing users revealed convergence among PEPFAR implementing partners in demands for data, data use infrastructure, and internal processes that engaged data users and producers (Table 3). PEPFAR implementing partners, not directly providing services, used both laboratory and clinical data to monitor laboratory operational processes and progress of HIV program implementation. Laboratory data were from OpenELIS, and clinical data were exported from the electronic health record for HIV clients into Microsoft Excel spreadsheets. These organizations had data management units that compiled and validated the data collected from the clinical laboratories in the implementing districts. The programmatic units then received the processed data and conducted data analyses to monitor or evaluate progress achievements and shortfalls. The analyses informed leaders in deciding how their organizations could support the clinical laboratories to address areas of improvement.

While 3 referral laboratories (11.5% of the 26 participants) used data to inform laboratory operations and clinical decision-making, 4 other laboratories (15.4%) used data only for laboratory operations (Table 3). Three participants (11.5%) from 2 national referral laboratories and one regional referral laboratory shared that they not only used operational data from OpenELIS to monitor nonconformities and improve sample flows and result validation, but they also actively contributed to clinical decision-making by examining data on testing results and whether a client was on time for a test. The 10 participants (38.5%) from the rest of the referral laboratories were most interested in laboratory operational data and viewed themselves as suppliers of data to the clinicians for making decisions. As an existing user noted, “we give [the clinician] his result so it’s a chain which we play a part in,” describing laboratory staff as data providers.

**Table 3.** Data demanded, data use infrastructure, and uses of the data demanded at the 2 types of organizations where most participants of this study worked in Côte d’Ivoire in 2021‐2022.

Themes	CDC^a^, PEPFAR^b^ implementing partners	Referral laboratories
What data were demanded	Laboratory data on VL^c^, EID^d^ Clinical data on active client case Pharmacy data on filled prescriptions	Data on laboratory operational process
What infrastructure supported data use	OpenELIS^e^ Clinical records (electronic health records for HIV clients; Excel spreadsheets)	OpenELIS
What issues were understood or addressed using data	Identify districts or laboratories with performance issues Track lost-to-follow-up clients for VL testing or treatment Identify clients with unsuppressed VL	Bottlenecks in sample flow Nonconforming samples Turnaround time Workload
What decisions or strategies were made using data	Follow-up with specific districts, laboratories, or clients	Improve workflow Follow-up with satellite laboratories about nonconforming samples

a CDC: US Centers for Disease Control and Prevention.

b PEPFAR: US President’s Emergency Plan for AIDS Relief.

c VL: viral load.

d EID: early infant diagnosis.

e OpenELIS: open-source enterprise level laboratory information system.

#### External Pressure

The participants from PEPFAR implementing partners and the Retro-CI laboratory, established and funded by the US CDC, shared that how they used data in decision-making was heavily influenced by PEPFAR and the MOH.

The participants from the laboratories in the public health system shared that their data use behavior already existed to support internal performance monitoring and address MOH data requirements. They would share their existing data with donors or partners in the areas that these external entities supported, eg, HIV testing and donated equipment. An existing user stated that they reported data to PEPFAR every month because they were supported by PEPFAR, “but it’s really not (PEPFAR) who influence our activity,” indicating that the laboratory maintained operational independence despite donor reporting requirements.

#### Data Quality

When asked how data quality issues affect how often they used data for decision-making, the participants shared that data timeliness had a more direct impact on the frequency of data use. All existing users who participated in the first FGD shared that they had internal processes to ensure the quality of the data in the dashboards. Four participants from the second FGD with potential users shared that, since they had technical validations, the data quality should be fine. However, one participant disagreed, stating that, despite the processes and units that were intended to ensure data quality, there were still missing data or poorly documented data on paper forms that decreased data quality.

### VL and EID Dashboards

#### Frequency of Using the Dashboards

Three existing users shared that they used the dashboards monthly; 2 said quarterly; and one said every 6 months because they were most interested in stock shortages, but they said that the dashboards were not up to date on stock. One existing user said they only used the dashboards on an ad hoc basis because the dashboards were not updated as frequently as they preferred.

#### Reason for Using the Dashboards

The existing users considered the dashboards useful tools for identifying and raising awareness of problems and important references for communicating with other laboratories. An existing user from a national referral laboratory felt excited and relieved that the dashboards were available because they provided direct evidence of laboratory performances, and there was no need to guess how the performances had been. An existing user at a PEPFAR implementing partner agency was interested in the performances of other similar organizations, which the dashboards showed, so that they could cross-compare and “boost our team to work more.”

After examining the dashboards briefly during the FGD and IDIs, potential users shared the reasons that would motivate them to use the dashboards were serving as a model to peers (“people outside the laboratory can copy us and seek to get in touch with us to see how we have achieved this” [IDI with potential user 5]); accessing information quickly for decision-making (“very quickly we know where [tasks] get stuck [in the workflow] and what quick decision to take.” [FGD potential user 5]); and monitoring progress toward targets (“good to know that we have feedback [from the dashboards] about our implementation of the national guidelines.” [IDI with potential user 1]).

#### Facilitators

All existing users were confident that they themselves and the colleagues who used the dashboards routinely at work were capable of using the dashboards. The FGD participants within each group converged that the design of the dashboards was nice, the colors were beautiful and distinct, and the layout was easily readable. Two existing users (of the 16; 12.5%) shared that the need for the dashboards was common across the PEPFAR implementing partners and the US CDC, so the motivation for using the dashboards already existed.

The participants shared that the relative advantages of the dashboards were simplicity and straightforwardness compared to other data analysis and visualization tools such as Microsoft Excel or Microsoft Power BI. Compared to static data spreadsheets, the visualizations on the dashboards showed a lot more information in an interactive way. The participants explained that, since the configurations and structures of the dashboard interface were predefined, accessing specific information was faster and easier for people who monitor performances routinely. One potential user at a regional referral laboratory thought that, compared to Excel, which could also visualize data, the dashboards were faster and more straightforward, and it was also a direct way to convey information to others who wanted to learn about the VL and EID activities of the laboratory or the region. One potential user and one existing user highlighted the benefit of an interactive tool that could quickly display information that they needed, for example, meaningful disaggregation by population group, whereas Excel spreadsheets were static. As one potential user noted, (“with a simple click, […]we do not need to go and redo the calculations again [in Excel] to be able to access the information”).

Although one existing user perceived Excel and Power BI as more versatile in analyzing data exported from OpenELIS, they acknowledged that the dashboards were “accessible” (IDI with existing user 4). Sometimes if she “had not finished analyzing [data exported from OpenELIS], [she] will take the information from the dashboards,” indicating that the dashboards provided faster access to analyzed information that was useful for decision-making.

In terms of market pressure, the existing users converged during the first FGD that they were aware of other organizations, especially PEPFAR implementing partners, that were using the dashboards. In the subsequent IDIs, an existing user shared that

*generally all the partners in Côte d’Ivoire use the dashboards, I exchange with colleagues in other organizations, and everyone refers to the dashboards*

[IDI with existing user 5]

Most potential users (9 out of 10; 90%) did not know of any organization that used the dashboards. The only participant who had prior knowledge came from PNLS, which oversees all HIV-related activities in the country.

#### Potential Barriers

Three existing users (of the 16; 18.8%) shared that, although they were able to use the dashboards without difficulty, they were concerned that new users might need internet connections and computers or tablets to access these online dashboards. Both groups of existing and potential users would like to have more information and training or coaching on using the dashboards. One existing user thought that training or coaching would be useful for new users to orient themselves around the layout and functions of the dashboards, noting that “[new users] may be impressed, but the use will be difficult, they need explanations and coaching.” Indeed, all but 2 potential users (80%) had not heard of the dashboards through any training or awareness session, making it the primary reason why they had not used them.

#### Suggestions for Improving the Dashboards

The most mentioned suggestion was updating the data displayed in the dashboards more frequently. Four existing users (25%) and 3 potential users (30%) suggested updating at least every other week, with more near–real-time updates being preferred. One potential user cautioned the others during the FGD about having an ideal world with near real-time data updates, since there was a natural trade-off between the speed of posting data and the necessary biological and technical validations in the laboratory operational process. Therefore, weekly updates might be the most ideal scenario that the dashboards could realistically achieve (FGD potential user 5).

The participants also suggested various information to add to the dashboards so they can meet the needs of the organizations better. Three existing users (18.8%) and 2 potential users (20%) would like to have more indicators about the laboratory operational process and service quality, eg, the number of noncompliant and rejected samples and testing validity. Information about when and why major disruptions in laboratory services happened was highly requested by all but 2 existing users to provide context around suboptimal performance. The potential user from PNLS would like the dashboards to extend to all laboratory testing related to people living with HIV rather than just VL and EID.

## Discussion

### Principal Findings

This study highlights the integral role of data-driven decision-making in HIV program implementation and laboratory operations in Côte d’Ivoire. The findings reveal that data are widely used across organizational levels to monitor performance, address challenges, and inform decisions, but application varies between referral laboratories and PEPFAR implementing partners. While some organizations focus on operational monitoring, others integrate data into clinical decision-making, reflecting differences in roles and priorities. While existing users valued the dashboards for tracking performance and benchmarking, potential users faced barriers related to access, awareness, and training. Concerns about the need for additional indicators suggest opportunities for improvement. Strengthening capacity-building and data integration could enhance the impact of the dashboards on decision-making. Articles that describe dashboards displaying HIV-related data are not uncommon [34-40]. This study not only describes the features of the VL and EID dashboards in Côte d’Ivoire but also explores contextual factors, dashboard complexity, hardware availability, and internet connectivity that influenced the dashboard user experience.

The participants not only recognized the importance of data but also reported concrete behaviors of using data in decision-making. The recognition and behaviors were shared by both leaders and other staff members in an organization. This data culture, especially among leadership, seemed to be more supportive than in some LMICs reported in other studies [41,42]. The finding in our study could reflect a sample of participants who highly valued data, since more than half of the participants were existing dashboard users and had monitoring and evaluation as one of their key responsibilities.

We observed a virtuous cycle starting from a supportive data use culture at PEPFAR partner organizations and clinical laboratories in Côte d’Ivoire, which made people willing to use the VL and EID dashboards in their work. MOH supervisors can use the dashboards to provide regular feedback and comparative results to health facilities and laboratories, showcasing to both the data producers and users how the submitted data were used and enhancing the value of data collection and use [43-45]. The perceived value of data ultimately fed back into a supportive data use culture.

The participants at clinical laboratories shared that they submitted data to PEPFAR implementing partners monthly in addition to the routine data collection and use process established by the MOH. This indicates parallel data systems due to donors’ data demands, which may have been burdensome and led service providers to view themselves only as data producers [4,46]. Having access to useful tools such as the VL and EID dashboards that visualize the data that the laboratories submitted might motivate them to assume the role of data users [47].

All participants reported that their organizations had processes to validate data or ensure data quality, which gave them confidence in the quality of the data they collected, reported, and used. Key data validation or quality assurance activities include using the data validation report in OpenELIS, the electronic laboratory information system, and conducting routine data quality assessments. Our prior research supports this impression by showing that OpenELIS, the electronic laboratory information system feeding data into the dashboards, improved data timeliness and completeness significantly [48]. However, since poor data quality remains a barrier to data-driven decision-making in LMICs [41], more targeted research is necessary to understand the correlation between data quality, users’ confidence in data quality, and actual data use behaviors.

The findings about how the study participants viewed the dashboards conformed with what other studies reported, but the perceived facilitators and barriers for using the dashboards were an addition to the evidence. Ease of use was one of the most assessed dashboard characteristics [21], and the study participants found the VL and EID dashboards simple and straightforward. Simple innovations have a higher likelihood of effectiveness due to their ability to enhance user satisfaction and shorten the learning curve [49-51]. For dashboards that intend to simplify the data synthesis and analysis process for users, low complexity is an important determinant of effectiveness.

The market pressure from peer organizations may have motivated existing users to use the dashboards. This phenomenon was especially present among the organizations that worked directly with PEPFAR. It would be interesting to explore among the potential users who work indirectly with PEPFAR if the same phenomenon would apply when some potential users start using the dashboards while others do not. Late adopters often face significant pressure to implement innovations, especially when competitors or colleagues have already adopted them; when others in the field are using an innovation, individuals and organizations may feel compelled to follow suit [52]. In order for market pressure to exist, potential users first must know about the innovation. Marketing the dashboards through training and awareness activities as tools for building an organization’s credibility could be necessary before market pressure can build.

The existing users, all based in Abidjan or other urban areas, were concerned that potential users in other regions of the country might not have the necessary equipment or Internet connection to access the online dashboards and that they might need training to learn to use the dashboards. It is important to ensure the precondition of necessary equipment is met for achieving the outcome of increased user pool in the future [53]. Providing training or a support hotline might also be useful strategies to engage potential users [54].

### Limitations

The FGDs and IDIs were carried out by I-TECH personnel who were involved in the development and implementation process of the dashboards, potentially leading to social desirability bias in reporting barriers or issues. However, since we explained to the participants that the study was formative rather than evaluative, the social desirability bias might not have manifested to a great extent. Other limitations include potential difficulties in recalling or misunderstanding the questions. To partially address these limitations, we provided explanations of the interview questions and encouragement to offer honest critiques and suggestions for improvement.

In addition, the study sample was purposively selected and not intended to be statistically representative of all existing or potential users of the dashboards. Since this was a qualitative study, we did not collect objective observations or produce quantitative estimates of the phenomena discussed by the informants. Thus, we were unable to assess how common particular experiences or opinions may have been. Future mixed methods research could help corroborate the qualitative insights reported by triangulating them with quantitative data on dashboard use patterns and implementation outcomes.

Finally, since a single study team member coded all transcripts, there is a possibility of subjective bias or limited interpretive diversity in the coding process. This risk was mitigated by independent review and validation of the thematic analysis by 2 additional team members.

### Conclusion

This study demonstrated that decision makers and program staff in Côte d’Ivoire’s HIV response all valued data-driven decision-making. They recognized the utility of the HIV VL and EID dashboards in monitoring performance and guiding decisions. While PEPFAR partners valued laboratory, clinical, and pharmacy data and used them in client- or program-level monitoring, laboratories demanded data on laboratory operational processes to improve workflow and quality control. These insights contribute to the broader understanding of effective data use and dashboard design in Côte d’Ivoire.

## Abbreviations

CDC: Centers for Disease Control and Prevention
CFIR: Consolidated Framework for Implementation Research
CNESVS: Comité National d’Ethique des Sciences de la Vie et de la Santé
EID: early infant diagnosis
FGD: focus group discussion
HIPAA: Health Insurance Portability and Accountability Act
I-TECH: International Training and Education Center for Health
IDI: in-depth interview
LMICs: low- and middle-income countries
MOH: Ministry of Health
OpenELIS: open-source enterprise-level laboratory information system
PEPFAR: United States President’s Emergency Plan for AIDS Relief
PNLS: Programme National de Lutte contre le Sida (National AIDS Control Program)
PRISM: Performance of Routine Information System Management
UNAIDS: Joint United Nations Programme on HIV/AIDS
VL: viral load
